# Disentangling the effects of circulating IGF-1, glucose, and cortisol on features of perceived age

**DOI:** 10.1007/s11357-015-9771-3

**Published:** 2015-04-16

**Authors:** Kelly van Drielen, David A. Gunn, Raymond Noordam, Christopher E. M. Griffiths, Rudi G. J. Westendorp, Anton J. M. de Craen, Diana van Heemst

**Affiliations:** 1 Department of Gerontology and Geriatrics, Leiden University Medical Center, PO Box 9600, 2300 RC Leiden, The Netherlands; 2 Unilever Discover, Sharnbrook, Bedfordshire, UK; 3 Dermatological Sciences, Salford Royal Hospital, University of Manchester, Manchester, UK; 4 Leyden Academy on Vitality and Ageing, Leiden, The Netherlands; 5 Netherlands Consortium of Healthy Aging (NCHA), Leiden, The Netherlands

**Keywords:** Skin aging, Glucose, Insulin-like growth factor 1, Cortisol

## Abstract

Circulatory levels of insulin-like growth factor (IGF-1), glucose, and cortisol have been previously associated with facial aging. However, as these serum measures are related, it is unclear whether their associations with skin aging occur independently from each other. We aimed to investigate whether the associations between serum IGF-1, glucose, and cortisol levels and perceived age/wrinkle grade occur independently of each other and whether these are mediated via skin wrinkling or via other skin aging features. Perceived age and skin wrinkling grade were assessed in a random sample from the Leiden Longevity Study with non-fasted (*N* = 579) and fasted blood sampling (*N* = 219). In our study population, a higher non-fasted IGF-1 level was associated with a lower skin wrinkling grade (*p* value = 0.014) and tended to associate with a lower perceived age (*p* value = 0.067), which was mediated for approximately 100 % by skin wrinkling. A higher non-fasted glucose level was associated with a higher perceived age (*p* value = 0.017), which was mediated for 51 % by skin wrinkling grade (*p* value = 0.112). A higher fasted cortisol level tended to associate with a higher perceived age (*p* value = 0.116), which was mediated for 29 % by skin wrinkling. Results remained similar when the serum measures were statistically adjusted for each other. Thus, the previously reported serum measures associate independently from each other with skin aging. IGF-1 is predominantly associated with perceived age by skin wrinkling, whereas cortisol and glucose also by other skin aging features.

## Introduction

Facial appearance, or perceived age, is composed of different facial features, including skin wrinkling, lip height, pigmented spots, and the nasolabial fold (Gunn et al. 2009; Nkengne et al. 2008). A higher perceived age (or older looking facial appearance) is associated with a higher risk of morbidity and mortality (Christensen et al. 2009). Multiple external factors associate with a higher perceived age, including a low social class, smoking, low body mass index, and a high sunlight exposure (Rexbye et al. 2006). However, studies conducted in twins suggest also a genetic contribution, and thus the contribution of intrinsic factors on a higher perceived age (Gunn et al. 2009; Shekar et al. 2005).

Previously, we reported that high levels of glucose and cortisol and low levels of insulin-like growth factor (IGF-1) were associated with a higher perceived age (Noordam et al. 2012, 2013a, b). In these publications, potential mechanisms have been postulated through which higher serum measures may lead to a lower or higher perceived age. IGF-1, which can be produced by dermal fibroblasts and keratinocytes of the stratum granulosum, promotes collagen expression and inhibits expression of matrix metalloproteinase-1 (MMP-1), a key enzyme involved in the break-down of the collagen matrix (Kilani et al. 2007; Rudman et al. 1997). Prolonged exposure to elevated glucose levels, as seen in diabetic patients, promotes premature cellular senescence in dermal fibroblasts and collagen cross-linking (Blazer et al. 2002; Dekker et al. 2009; Paul and Bailey 1996). Both affect the integrity of the collagen matrix. Chronically high serum cortisol levels, as seen, for example, in patients with Cushing syndrome, have deleterious effects, especially on skin tissue (Boscaro et al. 2001). As a result of the elevated cortisol levels, changes in various structures of the extracellular matrix may occur, such as suppression of hyaluronan synthase and a reduction in collagen mass (Autio et al. 1994; Averbeck et al. 2010).

However, although different mechanisms have been postulated for every of the three previously reported serum measures that associate with perceived age (Noordam et al. 2012, 2013a, b), independency of these associations from each other has not yet been examined in human population studies. Furthermore, the serum measures might affect different aspects of facial aging. For example, IGF-1 has been associated with perceived age but only through skin wrinkling (Noordam et al. 2013a). Whether this applies to the other serum measures has not yet been studied. Within the present study, we aimed to investigate the independency of the associations between serum glucose, IGF-1, and cortisol levels and perceived age and wrinkle grade. Furthermore, we assessed whether the serum measures were associated with perceived independence from skin wrinkling.

## Methods

### Study setting

The present study was conducted in the Leiden Longevity Study. This study was originally designed to identify genetic and phenotypic markers related to familial longevity. A comprehensive description of the study design and enrollment strategy has been published previously (Schoenmaker et al. 2006; Westendorp et al. 2009). In brief, a total of 421 long-lived Caucasian families were recruited, without selection on health conditions or demographics. Inclusion was performed when at least two long-lived siblings were still alive and fulfilled the age criteria of 89 years for men and 91 years for women. The offspring of these nonagenarian siblings were asked to participate as well, because they have a higher tendency to reach a very old age and there is a paucity of adequate controls at high age (Schoenmaker et al. 2006). Partners of the offspring were asked to participate as controls, because of their similar age and shared environment and lifestyle. The Leiden Longevity Study was approved by the Medical Ethical Committee of the Leiden University Medical Center. Written informed consent was obtained from all study participants.

### Study population

The research question was addressed using a cross-sectional study design. Between 2006 and 2008, 661 middle-aged participants (331 offspring and 330 partners) visited the research center for extensive examination, which included being photographed for the assessment of their perceived age and wrinkle grade. Of these 661 subjects, 234 participants (121 offspring and 113 partners) who lived nearby the research center (less than 45 min by car), were asked to come fasted at 08.30 a.m. for fasted blood sampling. Data from the non-fasted blood samples (samples between 2002 and 2006) were missing from 11 participants, and the wrinkle grading could not be ascertained in men with a beard (*n* = 37) or with a skin graft (*n* = 1). Furthermore, diabetic participants (*n* = 33) were excluded. Subjects had diabetes if they used glucose-lowering agents, had a non-fasted glucose level >11.0 mmol/L and/or had a medical history of diabetes. Analyses on non-fasted data were conducted in a population of 579 participants. Of the 234 participants with fasted data, participants using oral corticosteroids (*n* = 3) and participants with diabetes were excluded (*n* = 3). Wrinkle grading could not be assessed in men with a beard (*n* = 9), and these were therefore excluded as well. Therefore, the analyses of the fasted data were conducted in a population of 219 participants.

### Perceived age and wrinkle-grading assessments

The methods to determine a person’s perceived age and the grade of wrinkling have both been validated previously (Griffiths et al. 1992; Gunn et al. 2008, 2009). In brief, participants were asked to come to the study center without any makeup or hairstyling products. Facial photographs were taken from the front and at 45° with concealed hair and clothing. Based on these photographs, the perceived age was calculated by the average perceived age as assessed by 60 independent assessors. Assessors were predominantly British and had no previous age assessment experience. Although there was some variation in age and gender between the assessors, this variation has previously been shown to have a negligible effect on the mean age assessment it a large number of assessors are used (Gunn et al. 2008). The inter-rater reliability of the perceived age assessment had a Cronbach’s alpha of 0.99. The wrinkle grade was determined by the number and depth of fine and coarse wrinkles by means of frontal facial photographs and graded by two dermatologists. The skin wrinkling grade ranged from 0 to 9 points (Griffiths et al. 1992).

### Serum measurements

The levels of IGF-1 and insulin-like growth factor binding protein 3 (IGFBP3) were measured using a chemiluminescent immunometric assay on a Siemens Immulite 2500 analyzer (Siemens Healthcare Medical Diagnostics, Bad Nauheim, Germany). As a proxy of free IGF-1 in serum, we calculated the molar ratio of IGF-1 and IGFBP3 using the molecular masses of these proteins (7.5 kDa for IGF-1 and 28.5 kDa for IGFBP3). A higher ratio is indicative of a larger amount of free IGF-1 in serum. Glucose levels were measured with the Hitachi Modular P800 (Roche, Almere, the Netherlands). Cortisol levels were measured using a cortisol assay (ECLIA) on the Modular E170 (Roche Diagnostics, Mannheim, Germany).

### Co-variables

The following co-variables are considered: offspring/partner status, sex, chronological age, body mass index (BMI), smoking status, hypertension, and depression. Offspring/partner status refers to the possible inheritance of familial longevity (Schoenmaker et al. 2006; Westendorp et al. 2009). Chronological age refers to the age at which the facial photographs were taken. Weight and height, to calculate the BMI, were measured by research nurses at the study center. BMI was calculated by dividing weight (in kilograms) with height squared (in meters). Current smoking status (yes/no) was retrieved by lifestyle questionnaire. The diagnosis of hypertension was based on information retrieved from the general practitioner. The presence of a possible depression was based on pharmacy records (ATC code, N06A).

### Statistical analyses

Characteristics of the study population were assessed separately for participants from whom we had non-fasted and fasted serum measures.

We used linear regression models to study the associations between the non-fasted and fasted serum levels (IGF-1/IGFBP3 molar ratio, glucose, and cortisol) and perceived age and skin wrinkling. Participants were divided in tertiles concurring to their IGF-1/IGFBP3 molar ratio and level of glucose and cortisol. The estimated mean perceived age and wrinkle grade was adjusted for the following considered confounding factors: offspring/partner status, sex, chronological age, BMI, and current smoking. In a second analysis, we additionally included the skin wrinkling grade as a co-variable in the model, to assess whether the associations between the biomarkers and mean perceived age were mediated by skin wrinkling. In a sensitivity analysis, we additionally adjusted for antihypertensive drugs and antidepressants in the fasted analysis, since hypertension and depression have been associated with higher cortisol levels (Hammer and Stewart 2006; Tafet et al. 2001).

All analyses were conducted in a two-step approach. First, the associations between a serum measure and perceived age and skin wrinkling grade were assessed in separate models. Second, the association between a serum measure and perceived age or skin wrinkling was statistically adjusted for the other serum measures to test independency from each other. For example, the association between glucose and perceived age was additionally statistically adjusted for the IGF-1/IGFBP3 molar ratio. In the fasted study sample, also serum cortisol was additionally included in the statistical model.

To determine whether an observed association was independent from the skin wrinkling grade or independent from the other studied serum measures, we compared the beta estimates and tested mediation by skin wrinkling. Before adding the skin wrinkling grade as mediating factor to the statistical model, we tested for interaction between determinant and mediator by adding an interaction term of skin wrinkling grade and the serum measure to our linear regression model. When the interaction was not statistically significant, we calculated the percentage of the association explained by skin wrinkling.

All statistical analyses were performed using SPSS v.20 for Windows (SPPS Inc., Chicago, IL, USA). Two-sided *p* values below 0.05 were considered statistically significant.

## Results

### Characteristics of the study population

The general characteristics of the study population are presented in Table 1. On average, perceived age was lower than the chronological age. The group of non-fasted subjects did not differ from the group of fasted subjects regarding their chronological age, perceived age, wrinkle grade, and non-fasted levels of the IGF-1/IGFBP3 molar ratio and glucose, body mass index, and current smoking habits.

**Table 1:** Characteristics of the study populations

	Non-fasted cohort (*N* = 579)	Fasted cohort (*N* = 219)
Women (*N* (%))	315 (54.4)	117 (53.4)
Offspring (*N* (%))	298 (51.5)	113 (51.6)
Chronological age (years)	62.8 ± 6.8	63.3 ± 6.8
Perceived age (years)	59.5 ± 7.8	59.5 ± 7.9
Wrinkle grade (points)	4.6 ± 1.3	4.6 ± 1.3
Serum blood parameters		
Non-fasted IGF-1/IGFBP3 molar ratio	0.113 ± 0.03	0.113 ± 0.02
Non-fasted glucose (mmol L^−1^)	5.8 ± 1.1	5.7 ± 0.9
Fasted IGF-1/IGFBP3 molar ratio		0.105 ± 0.03
Fasted glucose (mmol L^−1^)		5.1 ± 0.5
Fasted cortisol (mmol L^−1^)		0.504 ± 0.1
Current smoking (*N* (%))	74 (12.8)	21 (9.6)
Body mass index (kg/m^2^)	26.4 ± 3.9	26.3 ± 3.8

All continuous date are presented as means with standard deviation, unless otherwise stated

Abbreviations: *IGF*-*1* insulin-like growth factor-1, *IGFBP3* IGF-binding protein 3

### Non-fasted IGF-1 and glucose levels

The associations between non-fasted serum levels and perceived age and skin wrinkling score are presented in Table 2. Participants in the lowest tertile of the IGF-1/IGFBP3 ratio tended to have a higher mean perceived age than participants in the highest tertile, although this was marginally not statistically significant (61.1 and 60.1 years, respectively; *p* value for trend = 0.067). Participants in the lowest tertile of glucose had a statistically significantly lower perceived than participants in the highest tertile (60.1 and 61.4 years, respectively; *p* value for trend = 0.017). The beta estimates (depicted as the average difference in outcome between subsequent strata) for non-fasted IGF-1/IGFBP3 and glucose and perceived age did not materially differ when both serum measures were included in one statistical model.

**Table 2:** Perceived age and wrinkle grade in tertiles of the IGF-1/IGFBP3 molar ratio and glucose (separate and combined model)—non fasted

	Tertiles of parameter							
	Separate model (only IGF-1/IGFBP3 or glucose)				Combined model (IGF-1/IGFBP3 and glucose)			
	Low	Medium	High	Beta (SE)^a^	Low	Medium	High	Beta (SE)^a^
IGF-1/IGFBP3 ratio								
*N*	193	193	193		193	193	193	
Range	0.05–0.101	0.102–0.121	0.122–0.23		0.05–0.101	0.102–0.121	0.122–0.23	
Perceived age (years)^b^	61.1 (60.3–62.0)	61.0 (60.1–61.9)	60.1 (59.2–61.0)	−0.49 (0.27)*	61.0 (60.2–61.9)	61.0 (60.1–61.9)	60.2 (59.3–61.1)	−0.42 (0.27)
Perceived age (years)^c^	59.9 (59.4–60.5)	60.2 (59.7–60.8)	60.0 (59.4–60.5)	0.01 (0.17)	59.9 (59.3–60.4)	60.2 (59.7–60.8)	60.0 (59.4–60.6)	0.05 (0.17)
Wrinkle grade (points)^b^	4.98 (4.79–5.16)	4.86 (4.67–5.05)	4.70 (4.51–4.89)	−0.14 (0.06)**	4.97 (4.78–5.15)	4.86 (4.67–5.04)	4.71 (4.52–4.90)	−0.13 (0.06)
Glucose								
*N*	197	195	187		197	195	187	
Range (mmol L^−1^)	3.3–5.2	5.3–6.1	6.2–10.5		3.3–5.2	5.3–6.1	6.2–10.5	
Perceived age (years)^b^	60.1 (59.2–61.0)	60.8 (59.9–61.7)	61.4 (60.5–62.3)	0.63 (0.27)**	60.2 (59.3–61.0)	60.8 (59.9–61.7)	61.3 (60.4–62.2)	0.58 (0.27)**
Perceived age (years)^c^	59.8 (59.2–60.3)	60.0 (59.5–60.6)	60.3 (59.7–60.9)	0.31 (0.17)	59.8 (59.2–60.3)	60.1 (59.5–60.6)	60.3 (59.7–60.9)	0.31 (0.17)
Wrinkle grade (points)^b^	4.75 (4.56–4.94)	4.87 (4.68–5.05)	4.95 (4.77–5.14)	0.09 (0.06)*	4.76 (4.57–4.95)	4.85 (4.66–5.04)	4.93 (4.74–5.11)	0.07 (0.06)

Data are presented as means with 95 % confidence intervals unless otherwise stated

*IGF*-*1* insulin-like growth factor-1, *IGFBP3* IGF-binding protein 3

*p value <0.10; **p value <0.05

^a^Beta estimated depicted as the average difference in outcome and standard error between subsequent strata

^b^Adjusted for offspring/partner status, gender, chronological age, BMI, and current smoking

^c^Adjusted for offspring/partner status, gender, chronological age, BMI, current smoking, and skin wrinkling

Participants in the lowest tertile of the IGF-1/IGFBP3 ratio had a statistically significantly higher wrinkle grade than participants in the highest tertile (4.98 and 4.70 points, respectively; *p* value = 0.014). Participants in the highest tertile of the serum glucose levels tended to have a higher wrinkle grade, but this was not statistically significant. The beta estimates remained similar in the combined model of the IGF-1/IGFBP3 ratio and serum glucose levels.

We observed no interaction between serum measures and wrinkle grade (*p* values >0.05). The association between the IGF-1/IGFBP3 molar ratio and perceived age attenuated after additional adjustment for wrinkle grade (*p* value for trend = 0.926; beta estimates, −0.49 versus 0.01). Furthermore, the association between serum glucose levels and perceived age attenuated after additional adjustment for wrinkle grade, but a trend remained (*p* value for trend = 0.112; beta estimates, 0.63 versus 0.31). Skin wrinkling mediated the associations between IGF-1/IBFBP3 molar ratio and glucose and perceived age by respectively 101 and 51 %. Beta estimates remained approximately similar when the IGF-1/IGFBP3 molar ratio and serum glucose levels were combined in one statistical model.

### Fasted IGF, glucose and cortisol levels

The associations between fasted serum levels of IGF-1/IGFBP3 molar ratio, glucose, and cortisol with perceived age and wrinkle grade are presented in Table 3. Overall, no statistically significant associations were observed between all three studied serum measures and perceived age and wrinkle grade. However, a high IGF-1/IGFBP3 ratio tended to associate with a lower perceived age and wrinkle grade, and a high fasted level of glucose and cortisol tended to associate with a higher perceived age. Furthermore, a low IGF-1/IGFBP3 ratio and high level of glucose tended to associate with a higher rate of skin wrinkling. The beta estimates did not materially differ after we added the serum measures into one statistical model.

**Table 3:** Perceived age and wrinkle grade in tertiles of the IGF-1/IGFBP3 molar ratio, glucose, and cortisol (separate and combined model)—fasted

	Tertiles of parameter							
	Separate model (only IGF-1/IGFBP3, glucose, or cortisol)				Combined model (IGF-1/IGFBP3, glucose, and cortisol)			
	Low	Medium	High	Beta (SE)^a^	Low	Medium	High	Beta (SE)^a^
IGF-1/IGFBP3 ratio								
*N*	73	73	73		73	73	73	
Range	0.05–0.093	0.094–0.113	0.114–0.19		0.05–0.093	0.094–0.113	0.114–0.19	
Perceived age (years)^b^	61.7 (60.1–63.3)	61.0 (59.4–62.6)	60.2 (58.6–61.8)	−0.72 (0.47)	61.4 (59.8–63.1)	60.8 (59.2–62.4)	60.1 (58.5–61.8)	−0.63 (0.47)
Perceived age (years)^c^	60.3 (59.3–61.4)	59.6 (58.6–60.7)	59.5 (58.5–60.5)	−0.41 (0.30)	60.3 (59.2–61.3)	59.6 (58.6–60.7)	59.4 (58.3–60.4)	−0.37 (0.30)
Wrinkle grade (points)^b^	4.96 (4.63–5.28)	4.96 (4.63–5.28)	4.80 (4.47–5.13)	−0.08 (0.09)	4.91 (4.58–5.25)	4.91 (4.58–5.24)	4.80 (4.46–5.14)	−0.07 (0.09)
Glucose								
*N*	81	67	71		81	67	71	
Range (mmol L^−1^)	3.7–4.8	4.9–5.2	5.3–6.7		3.7–4.8	4.9–5.2	5.3–6.7	
Perceived age (years)^b^	60.7 (59.0–62.4)	60.2 (58.5–61.9)	61.5 (60.1–63.0)	0.43 (0.46)	60.7 (59.0–62.4)	60.2 (58.5–61.9)	61.4 (60.0–62.9)	0.36 (0.46)
Perceived age (years)^c^	59.9 (58.8–61.0)	59.2 (58.1–60.3)	60.1 (59.2–61.1)	0.15 (0.30)	60.0 (58.9–61.1)	59.2 (58.1–60.3)	60.1 (59.1–61.0)	0.11 (0.30)
Wrinkle grade (points)^b^	4.80 (4.46–5.15)	4.88 (4.53–5.23)	4.98 (4.68–5.28)	0.08 (0.09)	4.79 (4.44–5.14)	4.86 (4.51–5.22)	4.97 (4.67–5.27)	0.07 (0.10)
Cortisol								
*N*	72	74	73		72	74	73	
Range (mmol L^−1^)	0.023–0.429	0.430–0.549	0.550–0.952		0.023–0.429	0.430–0.549	0.550–0.952	
Perceived age (years)^b^	60.2 (58.6–61.8)	61.2 (59.6–62.7)	61.6 (60.0–63.2)	0.73 (0.44)	60.1 (58.5–61.7)	60.9 (59.3–62.5)	61.4 (59.8–63.0)	0.67 (0.44)
Perceived age (years)^c^	59.6 (58.5–60.6)	59.5 (58.5–60.5)	60.5 (59.5–61.6)	0.52 (0.30)*	59.5 (58.5–60.6)	59.3 (58.3–60.4)	60.4 (59.4–61.4)	0.49 (0.29)
Wrinkle grade (points)^b^	4.77 (4.44–5.10)	5.05 (4.73–5.36)	4.89 (4.56–5.21)	0.05 (0.09)	4.75 (4.42–5.09)	5.02 (4.69–5.34)	4.86 (4.53–5.19)	0.05 (0.09)

Data are presented as means with 95 % confidence intervals unless otherwise stated

*IGF*-*1* insulin-like growth factor-1, *IGFBP3* IGF-binding protein 3

*p value <0.10

^a^Beta estimate depicted as the average difference in outcome and standard error between subsequent strata

^b^Adjusted for offspring/partner status, gender, chronological age, BMI, and current smoking

^c^Adjusted for offspring/partner status, gender, chronological age, BMI, current smoking, and skin wrinkling

When we additionally adjusted for the wrinkling grade (*p* values for interaction >0.05), beta estimates for the IGF-1/IGFBP3 molar ratio (mediation = 43 %) and glucose (mediation = 65 %) attenuated. A trend remained for the association between cortisol and perceived age (mediation = 29 %).

Beta estimates did not materially differ after additional adjustment for antihypertensive drugs and antidepressants.

## Discussion

The results of our study were twofold. First, we observed that the associations of the IGF-1/IGFBP3 molar ratio, glucose, and cortisol with perceived age were not materially different when analyzed separately or combined, which indicates independency from each other. Second, we observed that the association of IGF-1/IGFBP3 molar ratio was with perceived age was mediated primarily via skin wrinkling, whereas the associations of glucose and cortisol with perceived age were (also) mediated via other, untested, features of facial aging.

As the serum measures seemed to be independent from each other with respect to their association with perceived age and wrinkle grade, this indicates that different biological mechanisms are at play. High levels of IGF-1 have an inhibitory effect on fibroblast MMP-1 mRNA and protein expression and stimulate the expression of collagen (Paul and Bailey 1996). MMP-1 is a collagenase, which breaks down collagen. High levels of MMP-1, seen in elderly skin, result in collagen fragmentation and deterioration of skin structure and function (Fisher et al. 2009). These processes, normally inhibited by a high IGF-1 concentration, possibly enhance the formation of wrinkles. Chronically high levels of glucose, as seen in patients with diabetes mellitus, causes a higher degree of cross-linking in collagen and elastin via the formation of advanced glycation end-products and premature cellular senescence in dermal fibroblasts (Blazer et al. 2002; Dekker et al. 2009; Lan et al. 2009; Paul and Bailey 1996). Based on this, we postulate that high levels of glucose lead to insufficient elastin repair and reduced cellular resilience to molecular stresses in the skin and subcutaneous tissues. Prolonged exposure to elevated levels of cortisol, as is the case in, for example, Cushing syndrome, results in alterations in various structures of the face such as fat tissues. The influence of subcutaneous tissues on perceived age could drive the associations between cortisol and perceived age. Indeed, facial attractiveness in young women (before wrinkles are evident) is linked with cortisol levels in the blood (Rantala et al. 2013).

**Fig. 1:**
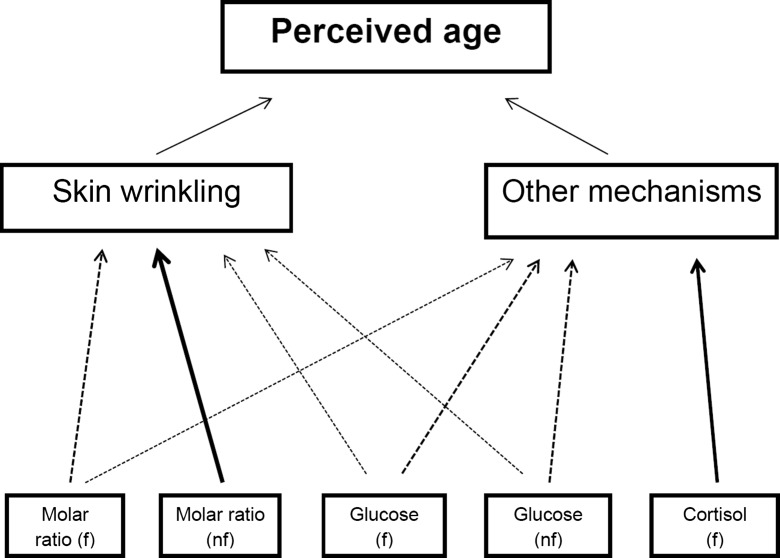
Schematic overview of the study findings. Abbreviations: *f* fasted, *nf* non-fasted. This figure present the possible routes through which non-fasted insulin-like growth factor (IGF)-1/IGF-binding protein (IGFBP) 3 molar ratio and non-fasted glucose and fasted IGF-1/IGFBP3 molar ratio, fasted glucose, and fasted cortisol influence perceived age. In our data, the association between perceived age and the non-fasted IGF-1/IGFBP3 molar ratio was mediated by skin wrinkling. The associations between the fasted-IGF-1/IGFBP3 molar ratio, non-fasted glucose, and fasted glucose were partially mediated by skin wrinkling and partially via other features, while the association between fasted cortisol and perceived age was mediated predominantly via other features than skin wrinkling

Beside skin wrinkling, several other potential mechanisms, such as sagging, have been described through which IGF-1, glucose, and cortisol could contribute to premature skin aging. Prior to this study, it was unclear for most measures what facial features are affected most by IGF-1, glucose, and cortisol. Figure 1 presents a schematic overview of our findings. To assess whether serum measures were associated with perceived age via skin wrinkling, we additionally statistically corrected the associations between the serum measures and perceived age for skin wrinkling grade. The association between cortisol and perceived age was minimally (29 %) mediated by skin wrinkling which suggests that cortisol drives skin aging predominantly via other features than skin wrinkling. For glucose, the association with perceived age was mediated to some extent by skin wrinkling (mediation was 51 and 65 %, respectively, for non-fasted and fasted measures). In contrast, results for IGF-1 were somewhat different for the non-fasted and fasted analysis. Results from the non-fasted analyses suggest that the IGF-1/IGFBP3 molar ratio affects skin aging predominantly via skin wrinkling (mediation = 101 %), whereas the results from the fasted analyses suggest that IGF-1/IGFBP3 molar ratio could also affect skin aging via other mechanisms (mediation = 43 %). A possible explanation for the discrepancy between the results obtained with non-fasted and fasted IGF-1/IGFBP3 molar ratio may be the difference in the years between when the blood samplings for measuring the IGF-1 and IGFBP3 levels were conducted, as the blood for measuring the non-fasted IGF-1 and IGFBP3 levels was drawn 4 years earlier than the blood for measuring fasted levels. This difference might be of importance as serum IGF-1 levels are known to decrease with increasing age (Juul et al. 1994). Moreover, different results obtained with the fasted and non-fasted samples could be due to measurements at a different time of the day and variation in time since the last meal. Although the circadian variation of IGF-1 and IGFBP3 is relatively small (Juul 2003), IGFBP3 is influenced by meal intake which could lead to a difference in the IGF-1/IGFBP3 molar ratio in the fasted samples compared with the non-fasted samples (Brand-Miller et al. 2005). A possible explanation for this discrepancy might also include the smaller sample size of the fasted group, which had less statistical power to detect associations of statistical significance. Taken together, still little is known about the specific changes to subcutaneous tissues that are affected by the serum measures. Therefore, more research is warranted.

This study had a few limitations. Because of the cross-sectional study design, we were not able to infer causality between the serum measures and perceived age and wrinkle grade. Facial photographs for assessing the perceived age and wrinkle grade were taken approximately 4 years later than the non-fasted blood samples. We are not able to judge the stability of the measurements during this period within the same individual. However, time between the two measurements might be in fact an advantage. The process of skin aging is not an acute effect, and thus the process of skin aging by the serum measures might be captured more accurately by the difference in time. Furthermore, the direction of effect with the fasted study sample was similar for the IGF-1/IGFBP3 molar ratio and glucose. Nevertheless, we should acknowledge that the fasted study sample is considerably smaller than the non-fasted study sample. This might be one of the reasons why some associations behaved differently in the non-fasted and fasted analysis after additional adjustment for skin wrinkling grade. Cortisol levels were measured at a single time point in the morning, although cortisol levels vary over the course of a day, with the mean peak just after awakening. Furthermore, evening or nocturnal cortisol levels are considered a better estimate of cortisol secretion than cortisol levels measured in the early morning (Dodt et al. 1994). In addition, the correlation between IGF-1 in serum and in facial skin is uncertain as IGF-1 can also be produced locally in many tissues. However, serum IGF-1 seems to correlate well with IGF-1 in facial sebum (Vora et al. 2008).

In conclusion, the data presented in this study suggests that IGF-1, glucose, and cortisol associate with perceived age independent of each other and may thus lead to skin aging via distinct biological mechanisms. IGF-1, and to a lesser extend also glucose, associate with perceived age through skin wrinkling, whereas cortisol may age the skin independently from skin wrinkling. Nevertheless, more research is warranted to assess through which specific facial features these serum measures affects skin aging.
